# ChromeBat: A Bio-Inspired Approach to 3D Genome Reconstruction

**DOI:** 10.3390/genes12111757

**Published:** 2021-11-03

**Authors:** Brandon Collins, Oluwatosin Oluwadare, Philip Brown

**Affiliations:** Department of Computer Science, University of Colorado, Colorado Springs, CO 80918, USA; bcollin3@uccs.edu (B.C.); philip.brown@uccs.edu (P.B.)

**Keywords:** Hi-C, 3D chromosome structure, bat algorithm, chromosome conformation capture, 3D genome

## Abstract

With the advent of Next Generation Sequencing and the Hi-C experiment, high quality genome-wide contact data are becoming increasingly available. These data represents an empirical measure of how a genome interacts inside the nucleus. Genome conformation is of particular interest as it has been experimentally shown to be a driving force for many genomic functions from regulation to transcription. Thus, the Three Dimensional-Genome Reconstruction Problem (3D-GRP) seeks to take Hi-C data and produces a complete physical genome structure as it appears in the nucleus for genomic analysis. We propose and develop a novel method to solve the Chromosome and Genome Reconstruction problem based on the Bat Algorithm (BA) which we called ChromeBat. We demonstrate on real Hi-C data that ChromeBat is capable of state-of-the-art performance. Additionally, the domain of Genome Reconstruction has been criticized for lacking algorithmic diversity, and the bio-inspired nature of ChromeBat contributes algorithmic diversity to the problem domain. ChromeBat is an effective approach for solving the Genome Reconstruction Problem.

## 1. Introduction

### 1.1. The Conformation Capture Assays

As DNA sequencing technology matures, so have questions surrounding how gene expression is functionally accomplished. It is well understood that genes require their associated regulators to function properly. However, DNA sequencing shows that a gene’s regulators may be many base pairs (bp) from the gene it regulates [1]. One experimentally proven mechanism to account for this disparity is the three-dimensional (3D) structure of the genome [2]. In particular, a gene’s regulator may be far in terms of linear base pairs, but in 3D space it could be quite local. Thus, it is imperative to understand a genome’s structure in 3D space as it is a mechanism for gene function.

There is a rich history of assay development to understand 3D genomic structure. Recently, the rise of genome interaction measurement techniques based on an “all versus all read-pair interaction profiling” [3,4] have enabled algorithmic approaches to reconstruct the genome. The first of these techniques, known as Hi-C [5], is summarized as follows: crosslink the chromatins using a fixative agent, digest the chromatin with a four or a six base cutter restrictive enzyme, apply biotin labels at the ends of the chromatins, relitigate the chromatins in dilute conditions, purify and shear DNA, and perform biotin pull-down [6]. Next, Next Generation Sequencing (NGS) technology is used for paired-end sequencing. The resulting reads are mapped to a reference genome and filtered. This step results in the creation of an Interaction Frequency (IF) matrix or contact matrix, representing relative levels of closeness of different portions of DNA called loci or bins. The length of the bins is called the resolution of the contact matrix. Hence, a bin with 1,000,000 base pairs has a resolution of 1 mb. The Hi-C method’s main innovation is that it can supply data across the entire genome, allowing 3D reconstructions at both the chromosome and genome-wide levels. Hi-C and related techniques are limited only by read depth and resolution restrictions presented by current sequencing technology. However, as NGS techniques steadily improve both in cost efficiency and throughput, Hi-C is poised to deliver genome-wide interaction data sets with ever increasing resolutions for bioinformatic analysis.

### 1.2. A Description of the Hi-C Experiment

The Hi-C technique is an all versus all technique for sequencing proposed in the novel study by Lieberman-Aiden et al. in 2009 [5], which efficiently pushed up the capabilities of 3C and 3C derived technologies. Hi-C uses the 3C template for crosslinking with formaldehyde to form links between physically adjacent DNA regions, followed by restriction digestion with specific restriction enzyme that is performed on the chromatin to digest chromatin into multiple DNA fragments. Next, the fragments are biotin-labeled by filling the fragments ends. The biotinylated fragments are then ligated followed by a biotin pull-down process where the DNA is purified and sheared. The biotinylated fragments are pulled down in this experiment with streptavidin beads in order to ensure that only the DNAs’ ligated ends are chosen to build the pair-end reads library and subsequent high-throughput sequencing. The sequenced pair-end reads are thereafter preprocessed through indexing or mapping, filtering, and noise removal to produce the contact or interaction frequency matrices, which is used today to provide explanations about the series of cell events such as genome folding, gene regulation, and genome stability and the relationship between regulatory elements and structural features in the nucleus of a cell.

### 1.3. The Genome Reconstruction Problem

In this work, we focus on using Hi-C data to solve the 3D genome reconstruction problem (3D-GRP). Formalized in [7], the 3D-GRP problem is defined as follows. First, a Hi-C experiment is performed, and a *contact matrix* is produced. A contact matrix is a square symmetric n×n matrix, where *n* is the number of loci at a given resolution. A solution to the 3D-GRP is set a (x,y,z) coordinates, one for each loci. A good solution will conform to the contact matrix from the Hi-C data. Approaches to solve this problem can fit into one of three categories. These are distance-based approaches, contact based approaches, and probabilistic approaches, which we briefly survey here [8].

Distance-based approaches feature two steps: first, the contact matrix must be converted to a *distance matrix*, and then an optimization technique is applied. By focusing on the first step, the contact matrix is converted to a distance matrix via an inverse relationship based on a constant α, called the conversion factor, typically in the range (0,3] [9]. Early approaches assumed an inverse relationship between distances, such as the 5C3D method developed in [10]. However, [11] demonstrated that the relationship between interaction frequency and distance can vary between experimental procedures and organisms illuminating the need for a principled method for picking α. One proposed solution [12] is to use microscopy data from FISH as a ground truth to assist in the interaction frequency to distance conversion process. Another approach [13] is to use a search algorithm to select a suitable α for each experiment.

Once the contact matrix has been converted to a distance matrix, the distance approaches proceed with optimization. One of the most popular choices [9] is to use a multidimensional scaling (MDS) approach [14]. This is the approach used in the classical 5C3D technique as well as more modern approaches such as miniMDS [15]. Another promising optimization process showcased in 3Dmax [16] involves formulating the problems in terms of maximum likelihood and solving it using an iterative technique such as gradient ascent. Other distance based methods include HSA [17], ChromeSDE [11], ShNeigh [18], Chromosome3D [19], and LorDG [20].

The second class of techniques is known as contact-based approaches. Unlike distance-based approaches, contact approaches derive a 3D structure directly from the Hi-C contact matrix. This is inherently advantageous as no assumption about a distance interaction frequency relationship needs to be made. The most straightforward of these approaches is known as MOGEN [21], which directly applies the gradient ascent optimization technique seeking to satisfy interaction thresholds given by the data. Purported to be robust against noise [8], it should be noted that noise and experimentally induced biases are highly nontrivial to handle. In order to mitigate this, contact based approaches have incorporated other sources of data such as fluorescence in situ hybridization (FISH) [22] as well as Lamina-associated Domains (LADs) [23].

The final class of genome reconstruction techniques is known as probability based methods. These methods function by defining a probability measure for contact frequencies. A major advantage for these methods is that uncertainty and bias in Hi-C data can be handled natively by a probabilistic method. Typically, probability-based approaches are ensemble techniques [8]. This entails that the method will output a population of models for which their averages are representative of the Hi-C data, which intuitively makes sense as Hi-C data is usually an average of many cells. The classical probabilistic method is known as MCMC5C [24] which generates an ensemble of models based on Markov Chain Monte Carlo sampling. Another example of a probability based approach is PASTIS [25].

Although these techniques vary greatly in performance, computational efficiency, and output file format, they all represent a solution to the 3D-GRP problem. Unfortunately, validating these solutions has itself proven to be challenging. For example, consider using a norm that measures the distance between the distance matrix and a proposed structure’s induced distance matrix to evaluate these solutions. Immediately, we must assume that some α exists such that the contact matrix can be converted to ground truth distances between all loci and that our solution finds it. Additionally, it is plausible that a more complex formula is better at converting interaction frequency to distance. Secondly, it would only be valid on single cell Hi-C data or else the objective function would seek an average of genomic structures that does not exist. In practice, the 3D-GRP solutions are validated using known genomic structures, other data such as FISH [8], or a simulated data set [26] where the ground truth structure is known. Thus, regardless of the quality of solution presented for the 3D-GRP, the validation that the procedure is generating genomic structures representative of actual cells remains an open question.

### 1.4. Motivating ChromeBat

**Figure 1:**
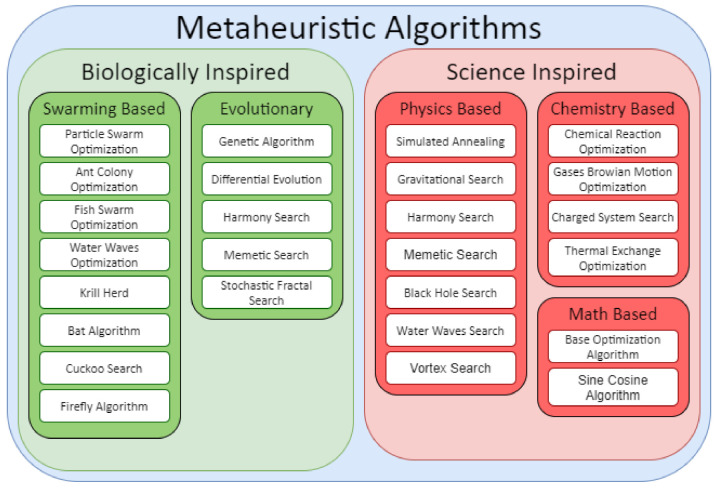
Classifications of Different Metaheuristic Algorithms. Compiled using information from [27,28,29,30,31,32,33].

The 3D-GRP has become an important problem in genomics and computational biology due to the structural impact on genomic function. In this work, we propose and explore a Bat Algorithm (BA) [34] based approach. The motivation for this approach is two-fold. First, existing approaches have been criticized for lacking algorithmic diversity [9]. Broadly speaking, the Bat Algorithm is a metaheuristic optimization algorithm, and an overview of metaheuristic algorithms can be observed in Figure 1. Notable applications of metaheuristic algorithms relative to 3D-GRP include the physics based simulated annealing (SA) [19,35] and the evolutionary genetic algorithms [36]. Specifically, the Bat Algorithm is a swarming algorithm that has seen no application on this problem, thus addressing the complaint of poor algorithmic diversity.

The second motivation for applying Bat Algorithm to 3D-GRP is that the algorithm uses intuitions from how bats hunt to navigate the fundamental tradeoff of exploration versus exploitation in optimization. Bats are largely blind predators that use echolocation to solve their objective of finding prey. They can alter their frequency and volume, where high frequency yields a short range but high-resolution picture and vice versa for low frequency. They typically begin their search at high volume but then lower it as they draw near their prey.

These intuitions are especially important due to the high dimensionality of the 3D-GRP. For example, at 1 mb, chromosome 1 in humans has approximately 250 loci which all have x,y,z coordinates resulting in approximately 750 parameters to optimize. From an optimization perspective, this is a high dimensional optimization problem, and the Bat Algorithm has been shown to be effective in image processing [37], which is a high dimensional domain. Thus, the Bat Algorithm will use the intuitions of how bats hunt in order to balance exploration versus exploitation to successfully optimize in a high dimensional context.

## 2. Materials and Methods

### 2.1. Loss Function

Let $A\in R^{n\times n}$ be a genome wide contact matrix obtained from a Hi-C experiment with *n* loci. We define genomic distance function between loci *i* and *j* as follows.

(1) $$d\left(i,j\right)=\frac{1}{A_{i,j}^{\alpha }}$$

From Equation (1), we define distance matrix matrix *D*. That is, $D_{i,j}=d\left(i,j\right)$ given contact matrix *A* and conversion factor α∈R.

By using *D*, we define the loss function as follows:

(2) $$L\left(S\right)=\sum_{i=1}^{n}\sum_{j=1}^{n}\left|D_{i,j}-\sqrt{{\left(x_{i}-x_{j}\right)}^{2}+{\left(y_{i}-y_{j}\right)}^{2}+{\left(z_{i}-z_{j}\right)}^{2}}\right|$$

where *S* is a proposed structure with *n*(x,y,z) coordinates, and $D_{i,j}$ is defined. ChromeBat utilizes the loss function presented in Equation (2) that has been used in other studies [1]. The loss function measures the difference between distance matrix *D* and the distance matrix induced by *S*.

### 2.2. Bat Algorithm for the 3D-GRP

**Figure 2:**
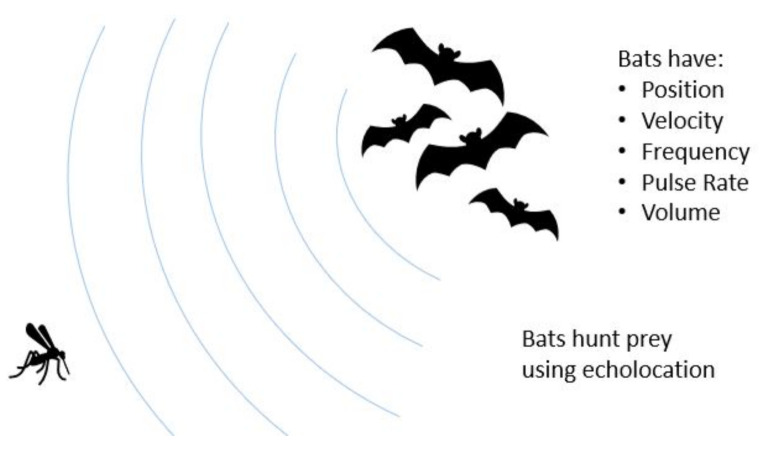
Visualization of the Bat Algorithm. The Bat Algorithm is inspired by the natural hunting behavior of bats. The algorithm captures this by giving each bat a the set of variables pictured on the right. These variables and their interaction are formalized in Section 2.2.

**Figure 3:**
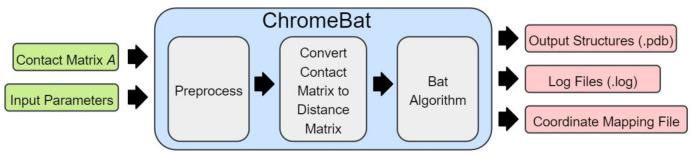
Visualization of ChromeBat’s Pipeline from Contact Matrix to Predicted Structure. Further description of the preprocessing and optimization steps can be found in Section 2.2 and Section 2.3, respectively.

Figure 2 provides the illustration of how the bat hunts a prey using echolocation. It also illustrates visually the properties (or variables) that bats possess for natural hunting. We now provide the full description of BA and its implementation in ChromeBat. Note that the following discussion describes how the BA is implemented in ChromeBat, which varies slightly from what was originally proposed. The full pipeline for our algorithm from input to predicted structures can be observed in Figure 3. The list of hyperparameters for the ChromeBat algorithm is described in Section 2.4.

First, we initialize *k* bats, where each bat *i* “knows” three locations, $S_{i}$, $S_{i}^{*}$, and $S^{*}$, representing a bat’s current location, its personal best known location, and the global best location, respectively. Note that all of these vectors and velocity vector $V_{i}$ are all in $R^{3n}$, where *n* denotes the number of loci from the Hi-C experiment. We initialize $S_{i}^{*}$ randomly, $V_{i}$ as the the 0 vector, and $S^{*}$ using Equation (7). The algorithm then proceeds for *T* iterations.

Each loop of the algorithm begins by the bats updating their current location $S_{i}$ with the following equations:

(3) $$f_{i}=f_{min}+\left(f_{max}-f_{min}\right)u\left(0,1\right)$$

(4) $$V_{i}=V_{i}+\left(S_{i}^{*}-S^{*}\right)f_{i}$$

(5) $$S_{i}=\left\{\begin{array}{cc} S_{i}^{*}+V_{i} & \mathrm{with probability}1-r\\ S^{*}+pG\left(0,1\right) & \mathrm{else} \end{array}\right.$$

where G(μ,σ) denotes a vector of 3n values where each one is sampled from a normal distribution with mean μ and standard deviation σ, and u(a,b) denotes a value selected uniformly at random from the interval [a,b]. Note by the random valued condition in Equation (5), the bats update their position with one of two methods. If they decide not to pulse (corresponding to probability 1−r), then they will select a random frequency in $\left[f_{min},f_{max}\right]$ as per Equation (3) and then use this value to randomly adjust their velocity $V_{i}$ in Equation (4). This velocity adjustment is based on a bats current best location $S_{i}^{*}$ and global best location $S^{*}$, guiding bats in a hopefully well selected direction. Then, the bats who did not pulse will use their newly updated velocity in order to update their current position $S_{i}$. On the other hand, if a bat chooses to pulse, invoking the second case in Equation (5), then the bat will teleport to the global best known location $S^{*}$ and take a random walk scaled by hyperparameter *p*. It can be observed that a high *r* corresponds to a bat who pulses with high probability and vice versa for low *r*.

Once bats have updated their location $S_{i}$, they decide whether or not to accept their new solutions to the 3D-GRP according to following equations:

(6) $$S_{i}^{*}=\left\{\begin{array}{cc} S_{i} & \mathrm{with probability}a\mathrm{if}L\left(S_{i}\right)\geq L\left(S_{i}^{*}\right)\\ S_{i}^{*} & \mathrm{else} \end{array}\right.$$

(7) $$S^{*}\in \underset{i}{arg min}\left(L\left(S_{i}^{*}\right)\right)$$

where *L* is the loss function defined in Equation (2). The conditions on whether a bat accepts its current solution $S_{i}$ are given in the top case of Equation (6) and can be interpreted as follows. For a solution $S_{i}$ to accepted, it must be a better solution than the currently accepted solution $S_{i}^{*}$, that is, $L\left(S_{I}\right)<L\left(S_{i}^{*}\right)$. Additionally, the bat’s volume *a* is used to introduce randomness in whether a bat accepts solutions frequently. From Equation (6), it can be observed that a high *a* means a bat will accept new solutions with high probability. The final step of the algorithm is to reapply Equation (7) to select the new global best solution $S^{*}$. After all *T* iterations are complete, the algorithm terminates and returns $S^{*}$.

**Figure 4:**
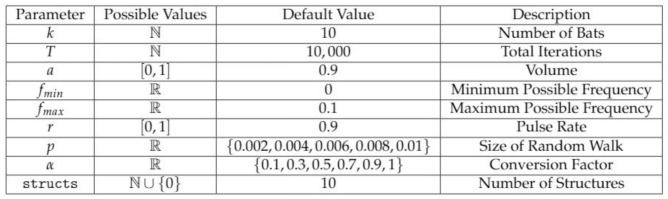
Hyperparameters in ChromeBat. Note that in the original work, certain variables such as *r* and *a* were proposed to change as the algorithm progressed. In ChromeBat, they are constant throughout. For more information about the default value selection, see Section 2.4.

Now that the BA has been discussed in detail, we provide an overview of the pipeline ChromeBat uses, depicted in Figure 3. First, ChromeBat is called in Python 3 and passed a HiC data file and a parameter file which contains all parameters given in Figure 4. Then, the preprocessing steps discussed in Section 2.3 are performed on the HiC data. If *p* or α passed multiple values, then ChromeBat will use BA to produce a structure for each combination of (p,α) and evaluate them by using dSCC. Particularly, α is used to convert the HiC contact matrix into a distance matrix via (1), and *p* is a perturbation parameter used in BA as discussed above. Note that dSCC is evaluated relative to the distance matrix *D* induced by α that was used to convert the matrix. The combination that produced the highest dSCC is selected, and then the algorithm generates structs structures using the BA with these parameters.

### 2.3. Preprocessing

Due to the noisy and inconsistent nature of HiC data, ChromeBat automatically performs two preprocessing steps. First, ChromeBat removes any row and column *i* if both are all zero. That is, if the *i*th row and *i*th column are both all zero, then ChromeBat will remove both the row and the column retaining a square matrix. ChromeBat additionally outputs a coordinate mapping file which indicates how the original loci map to the new loci.

Secondly, ChromeBat performs an adjacency normalization step on the contact matrix. Recall that in contact matrix *A*, the contact counts of loci *i* to its adjacent loci i+1,i−1 are given by $A_{i,i+1},A_{i,i-1}$. Occasionally, in HiC data, it happens that $A_{i,i+1}=0$ or $A_{i,i-1}=0$ for some loci *i*. This is misleading as these loci are near each other by definition. To remedy this, we average all nonzero adjacent loci and set each zero adjacent loci to be this average. Note that this step is only used for optimization purposes, and the evaluations described in Section 2.5 are performed by using the contact matrix without this step.

### 2.4. Hyperparameter Selection

As observed in Figure 4, ChromeBat features many hyperparameters. To select default values for the algorithm, we conduct a series of experiments on the simulated helical structure data presented in [17]. More details on this data set can also be found in Section 2.6, and all experiments are performed on the 90% coverage version.

Initially, we take $f_{min}=0$ as it is intuitive by Equation (3) that bats should select frequency uniformly between 0 and $f_{max}$. Then, we perform four searches across with $f_{max}$, *p*, *r*, and *a* at the same time. We find in all of these searches that α=0.5, so we may fix it for future searches. Additionally, we find the greatest distance Spearman Correlation Coefficient (dSCC) result occurred at p=0.9 and a=0.9; thus, we fix these parameters.

With α known, we conduct an experiment searching across *T* and *k* as these parameters solely determine the runtime of the algorithm. We find k=10 and T=10,000 sufficient, noting the differences in dSCC between runs with greater *T* and *k* are negligible. We also notice from this experiment that no matter how large *T* and *k* are, the algorithm appears to become“stuck” sometimes. To remedy this, we introduce another hyperparameter structs that represents how many structures the algorithm should generate for consistency. We take structs=10 to balance computation time and performance of the algorithm. Further discussion of this parameter can be observed in Section 3.3.

Finally, we carry out a search across $f_{max},p$, where we take generate 10 structures per parameter combinations due to concerns about the methods consistency from the previous search. To ensure consistency, we use the average dSCC across the 10 generated structures and the most consistent and best performance from $f_{max}=0.1$ and p=0.002. Thus, we fix $f_{max}=0.1$ but we find impressive performance across all *p* values searched {0.002,0.004,0.006,0.008,0.01}. Thus, for the default behavior of the method, we include a search across α and *p*.

### 2.5. Evaluation

To validate our method, we used the distance Spearman Correlation Coefficient (dSCC) metric. The equation for this metric is given by the following:

(8) $$dSCC\left(D_{S},D_{K}\right)=\frac{\sum_{i=1}^{n}\left(X_{i}-\bar{X}\right)\left(Y_{i}-\bar{Y}\right)}{\sqrt{\sum_{i=1}^{n}{\left(X_{i}-\bar{X}\right)}^{2}\sum_{i=1}^{n}{\left(Y_{i}-\bar{Y}\right)}^{2}}}$$

where $D_{K}$ is a set containing all unique distance measures between loci from the Hi-C experiment. That is, $D_{K}$ has an element for each uniquely defined entry of *D* (one entry for $D_{i,j}$ and $D_{j,i}$). Let $D_{S}$ be a set of all corresponding distance measures from the generated structure, particularly that $D_{S}$ contains the distance measure between loci i,j only if $D_{i,j}$ is defined. Then let *X* and *Y* be ranked the variables corresponding to $D_{S}$ and $D_{K}$, respectively, and let $\bar{X},\bar{Y}$ refer to the mean of the ranked variables. Recall *n* refers to the total number of bins or regions in the Hi-C data. Note that when we evaluate dSCC, the $D_{S}$ set is induced from distance matrix *D* defined by (1) using the conversion factor α that the method used.

### 2.6. Datasets

In order to demonstrate the effectiveness of ChromeBat, we compared the method with techniques studied in the literature on two cell lines and a simulated data set. The first cell line is GM06990, and it was originally sequenced by Lieberman et al. [5] and processed and normalized in [18]. The GM06990 cell line was restricted both with HindIII and Ncol enzymes and downloaded from https://github.com/fangzhen-li/ShNeigh/, accessed on 15 August 2021. We consider this cell line both at 1 mb and 500 kb resolutions.

The second cell line is the normalized GM12878 cell line [38] downloaded from GSDB [39]. The normalization method used is the Knight–Ruiz [40]. This cell line contains 4.9 billion pairwise contacts at map resolution 950 bp. It was gathered from Human GM12878 B-lymphoblastoid cells and aggregated from nine cultures.

To ensure rigor, we also evaluate ChromeBat on a simulated contact map created by Adhikari et al. [19] from a theoretical 3D model structure representing yeast’s chromosome 4 at 50 kb [41]. This structure has 610 loci, and critically the ground truth structure is known. This allows comparisons to a known ground truth instead of an inferred structure (that is, via (1)). This analysis confirms ChromeBat’s ability to make structures that are representative of true structures. The data can be downloaded from Adhikari et al. [19].

In addition to assessing ChromeBat’s performance on real and simulated data, we tune its hyperparameters by using simulated data from [17]. The data were constructed by simulating a regular helical structure and deriving contacts maps at a desired signal coverage level. A signal coverage level merely denotes what percentages of entries in the contact matrix are non-zero. Zhang et al. [17] derived contact matrices at a desired signal coverage level by assuming that the contact matrix satisfies a Poisson distribution of a power law based on the actual distances. Then, they provide and test their methods on coverage levels of 90%, 70%, and 25%. The motivation for the simulated approach is that genome-scale ground truth exists for any genome reconstruction problem. Due to this, we use the simulated data set for hyperparameter selection of our model.

## 3. Results

### 3.1. Comparison with Metaheuristic Methods

In the literature, we have found six metaheuristic methods to compare our method against. These include the following: Gen3D [36], PGS [42], Chrom3D [23], 3D-GNOME [43], Chromosome3D [19], and HSA [17]. Of these, three are distance based (3D-GNOME, Chromosome3D, and HSA), two are contact based (Gen3D and Chrom3D), and one is probability based (PGS).

Among these methods, Gen3D utilizes a genetic algorithm approach while the rest of the approaches are based on Simulated Annealing (SA). Unfortunately, PGS, Chrome3D, gen3D, and 3D-GNOME all require more input data in addition to the contact matrices; thus, we could not compare against them. Regardless, we compare ChromeBat on GM06990 and GM12878 cell lines as discussed in Section 2.6 with HSA and Chromosome3D.

**Figure 5:**
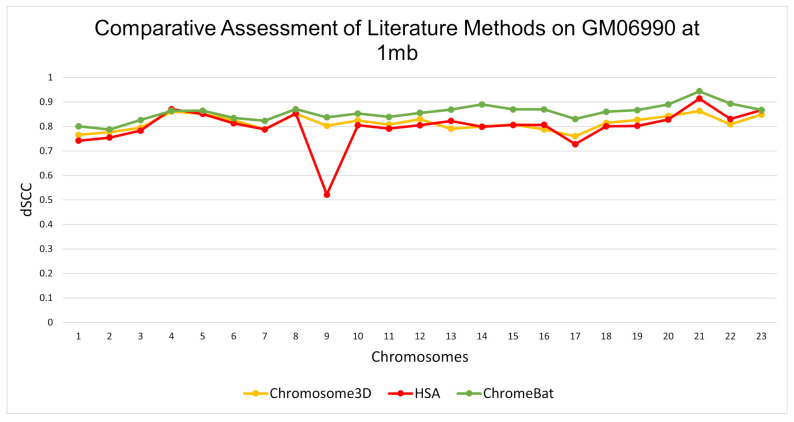
Comparative Assessment of Metaheuristic Methods on GM06990. A comparison of the distance Spearman Correlation Coefficient (dSCC) between metaheuristic methods Chromosome3D, HSA, and ChromeBat. This experiment is performed on the first 23 chromosomes of the GM06990 cell line at 1 mb resolution using restriction enzyme HindIII.

**Figure 6:**
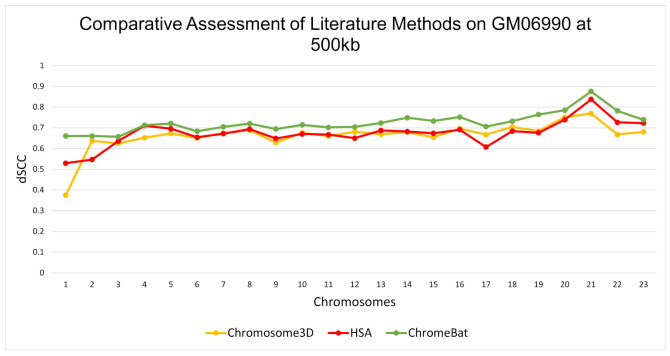
Comparative Assessment of Metaheuristic Methods on GM06990. A comparison of the distance Spearman Correlation Coefficient (dSCC) between metaheuristic methods Chromosome3D, HSA, and ChromeBat. This experiment is performed on the first 23 chromosomes of the GM06990 cell line at 500 kb resolution using restriction enzyme HindIII.

In the GM06990 cell line at 1 mb and 500 kb observed in Figure 5 and Figure 6, ChromeBat has the highest dSCC across almost all chromosomes evaluated. In particular, ChromeBat outperforms all other metaheuristic methods by at least 5% in chromosomes 14–18.

### 3.2. Comparison with Existing 3D-GRP Methods in Literature

In order to verify that ChromeBat is not only competitive among metaheuristic methods, we compared it against five literature methods on the GM06990. The methods we compare against include 3Dmax [16], HSA [17], ShNeigh2 [18], Chromosome3D [19], and LorDG [20].

**Figure 7:**
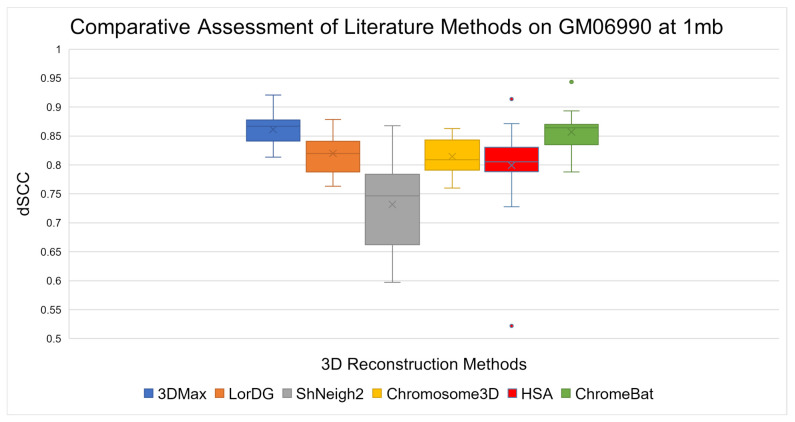
Comparative Assessment of Literature Methods on GM06990. A comparison of the Spearman Correlation Coefficient between literature methods 3Dmax, LorDG, ShNeigh2, Chromosome3D, HSA, and ChromeBat. This experiment is performed on the first 23 chromosomes of the GM06990 cell line at 1mb resolution using the HindIII restriction enzyme. That is, in each box plot, there is one data point per chromosome that represents the method’s dSCC performance on that chromosome.

The results on the GM06990 cell line can be observed in Figure 7. Overall, ChromeBat performs competitively across the board within a close margin of 3Dmax on every chromosome. It can be observed that ChromeBat has a similar mean performance to the best method by mean and 3Dmax. Additionally, ChromeBat achieved the overall single highest dSCC score on all chromosomes.

Furthermore, in order to verify that there is statistical difference between the methods, we applied the Mann–Whitney test to the methods. This test is used to validate or reject the null hypothesis that the two underlying distributions between two sets of observations are the same. We use each method to generate 35 independent structures on Chromosome 1 and treat their dSCC score as observations. Then, in Table 1, we conduct the statistical test between each set of observations. The value in the table is the *P* value of the test, which is interpreted as the confidence in which the null hypothesis is correct, where the null hypothesis is that the distributions underlying the observations are the same. As observed in the Table, the null hypothesis can be rejected between all methods, confirming the fact the methods have distinct performances.

### 3.3. Robustness

In this section, we conduct a series of experiments to verify that ChromeBat is performing on more than just the GM06990 cell line derived using the HindIII restriction enzyme. We begin by comparatively evaluating ChromeBats performance on the GM06990 cell derived from both the HindIII and the Ncol restriction enzymes. We further validate its performance by using FISH data on Chromosome 14 for both restriction enzymes. We proceed by evaluating the consistency and performance of Chromebat’s structures on the GM12878 cell line. To conclude, we evaluate ChromeBat and literature methods on the theoretical yeast chromosome 4.

**Figure 8:**
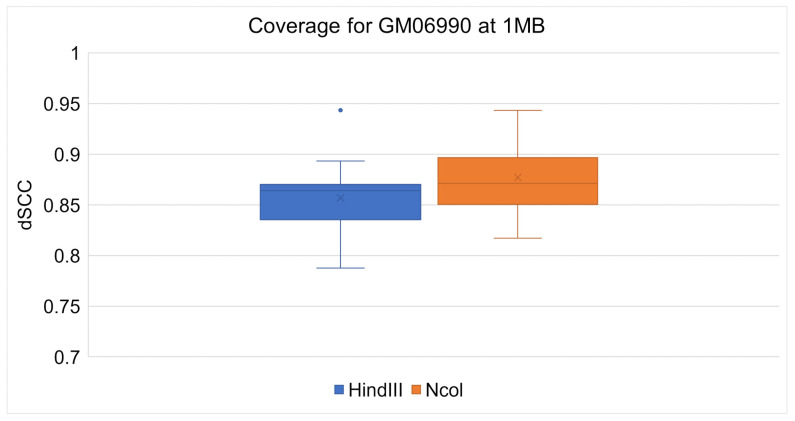
Chromebat’s Performance on the GM06990 at 1 mb for both restriction enzymes HindIII and Ncol.

**Figure 9:**
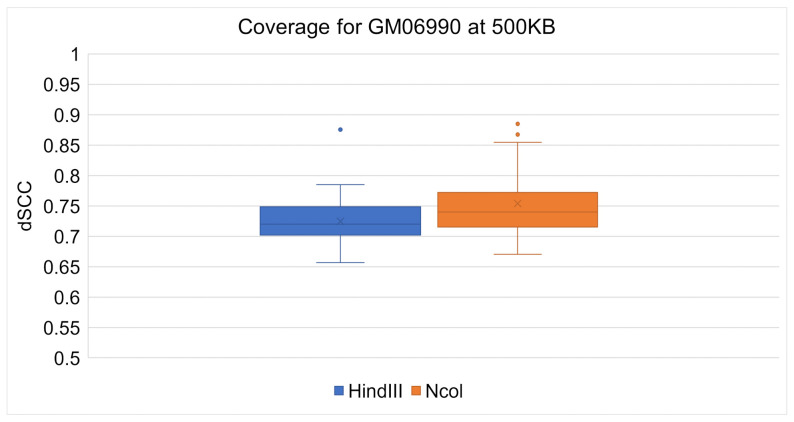
Chromebat’s Performance on the GM06990 at 500kb for both restriction enymes HindIII and Ncol.

ChromeBat’s comparative performance between the Ncol and HindIII restriction enzymes can be seen at 1 mb in Figure 8 and at 500 kb in Figure 9. It can be observed at both resolutions that ChromeBat performs better on Ncol then HindIII by every metric a box plot provides. Thus, we conclude that ChromeBat is robust to the restriction enzyme in the GM06990 cell line.

In order to further validate the robustness of ChromeBat relative to the compared methods, we conduct FISH validation on all compared methods. We utilize FISH data on chromosomes 14 at 1 mb by using both the HindIII and Ncol restriction enzymes. Chromosome 14 in the GM06990 cell line was FISH probed at four loci in [5]. These loci (L1,L2,L3, andL4), were gathered from consecutive positions in terms of base pairs but alternating between chromosome compartments A and B. In particular, (L1,L3) belongs to compartment A and (L2,L4) belongs to compartment B. Thus, for our generated structure to be consistent with the FISH data, we require (L1,L3) to be closer than (L1,L2) as well as (L2,L4) to be closer together then (L2,L3).

**Figure 10:**
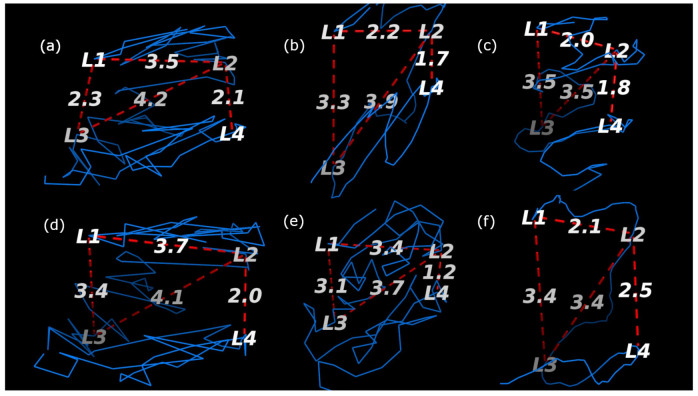
Fish Data Validation on Chromosome 14 from GM06990 litigated with HindIII at Resolution 1 mb. The blue line denotes the generated structure. The four probes (L1,L2,L3, and L4) from [5] are labeled on the chromosome, and the important distances between them have been labeled by red dashed lines. The methods used to generate each structure are as follows: (**a**) ChromeBat, (**b**) LorDG, (**c**) HSA, (**d**) 3DMax, (**e**) Chromosome3D, and (**f**) ShNeigh.

**Figure 11:**
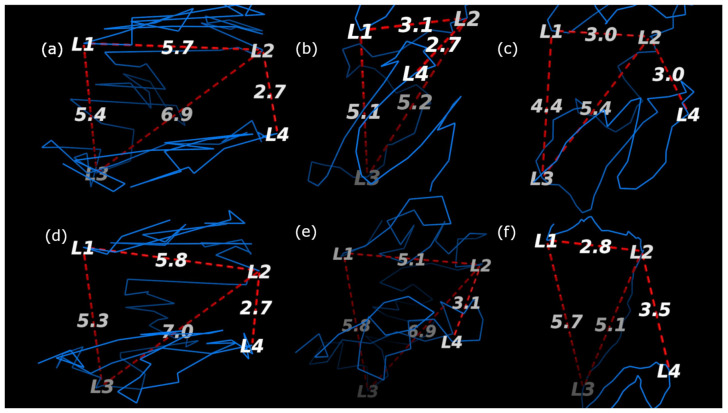
Fish Data Validation on Chromosome 14 from GM06990 litigated with Ncol at Resolution 1 mb. The blue line denotes the generated structure. The four probes (L1,L2,L3, and L4) from [5] are labeled on the chromosome, and the important distances between them have been labeled by red dashed lines. The methods used to generate each structure are as follows: (**a**) ChromeBat, (**b**) LorDG, (**c**) HSA, (**d**) 3DMax, (**e**) Chromosome3D, and (**f**) ShNeigh.

The structures that each method generated are visualized with the loci labeled in Figure 10 and Figure 11 for the HindIII and Ncol restriction enzymes, respectively. The results are summarized in Table 2 where it can be seen that only ChromeBat and 3Dmax are completely consistent with the FISH data. The remaining methods, Chromosome3D, LorDG, HSA, and ShNeigh, all struggled to ensure that the compartment A loci, (L1,L3), are closer together then the consecutive loci (L1,L2). This analysis and visualization was performed with PyMol [44].

In Section 2.4, we note that the method appears to struggle with consistency under certain parameters. We, therefore, investigated this phenomenon on a different cell line, GM12878, as described in Section 2.6. We performed two experiments on the GM12878 cell line using the parameters specified in Figure 4 with the exception that we take structs=35 and fix p=0.002 in the first experiment and p=0.004 in the second. Note that in this experiment, we plot every structure ChromeBat generated in contrast to earlier plots where the performance of ChromeBat is taken as the best structure generated.

**Figure 12:**
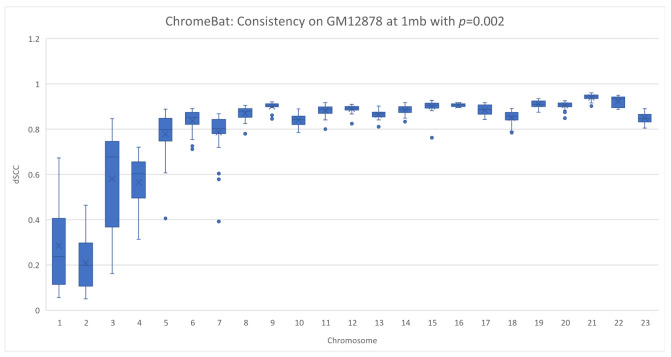
Consistency of ChromeBat on GM12878 with *p*=0.002. This is a consistency experiment done on GM12878 where hyperparameters from Figure 4 with the exception of the number of structures, structs, is 35 and p=0.002.

**Figure 13:**
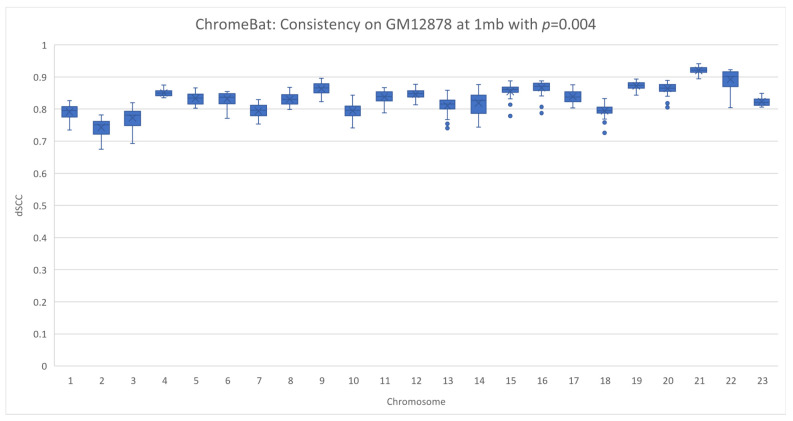
Consistency of ChromeBat on GM12878 with *p* = 0.004. This is a consistency experiment done on GM12878 where hyperparameters from Figure 4 with the exception of the number of structures, structs, is 35 and p=0.004.

The results are shown in Figure 12 and Figure 13. Visually, on the lower chromosomes with p=0.002 ChromeBat displays high variability in the performance between runs with the same parameters. However, simultaneously p=0.002 produces more consistent and better structures on the later chromosomes than the p=0.004 experiment, despite its poor performance on chromosomes 1–6. To formalize the fact that the dSCC distributions or the quality of structures generated are distinct, we carry out the Mann–Whitney U test shown in Table 3. This test is used to validate or reject the null hypothesis that the two underlying distributions between two sets of observations are the same. To perform this, we treat each dSCC value as an observation and test whether the structures generated with p=0.002 and p=0.004 indeed have a different underlying distribution. As observed in Table 3, the *P* value determines the confidence at which we can say the distributions are same. It can be observed that for all chromosomes, except chromosome 7, we are at least 95% confident the underlying distributions are not the same. Thus, altering parameter *p* indeed changes the quality of structure the method generates, justifying the need to search over *p*.

This reinforces the need to search across both α and *p*. Furthermore, it shows that when the parameters of ChromeBat are well tuned, it can produce consistent and performant structures. It is then also important to generate multiple structures with the same parameters after the search is performed, as the consistency of the produced structures reveals how well the hyperparameters are suited to the particular problem instance at hand.

**Figure 14:**
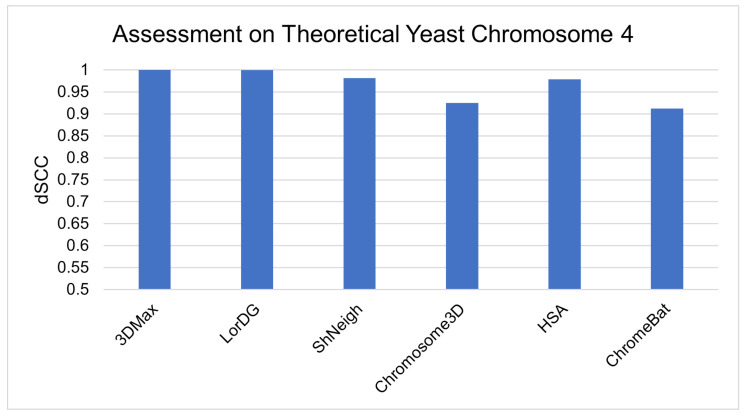
Comparative evaluation of methods on simulated yeast data.

Finally, we evaluate ChromeBat and the literature methods on theoretical yeast chromosome 4 data [19,41]. The advantage of simulated data is that the ground truth is known [41]. Thus, our method’s ability to recreate real structures can be better assessed. The results can be observed in Figure 14, where all methods can be seen to have strong performance with >0.9 dSCC.

## 4. Discussion

As highlighted in [9], the 3D-GRP lacks algorithmic diversity in general; however, as ChromeBat is a metaheuristic approach, we restrict our attention to algorithmic diversity among metaheuristic algorithms. We found six metaheuristic methods in the literature; however, out of those, five are based on Simulated Annealing and one on the Genetic Algorithm. Figure 1 highlights this shortcoming categorically. It can be observed that these methods only represent two categories of metaheuristic algorithms: evolutionary and physics based. Thus, ChromeBat is the first representative of the swarming-based methods, and many categories of metaheuristic algorithms are not studied on this problem.

Among metaheuristic algorithms, the need for more algorithmic diversity in the 3D-GRP can be observed in Section 3.1. In particular, the comparison on GM06990 given in Figure 7 at 500 kb showcases that ChromeBat gives state-of-the-art performances among metaheuristic methods. Considering how a few metaheuristic methods are deployable on raw Hi-C data and poor characterization of what makes a method perform well on a given dataset, ChromeBat contributes diversity to a less-studied class of the 3D-GRP methods.

The importance of algorithmic diversity can be observed in a broader scale in Section 3.2. In particular, on GM06990 shown in Figure 7, ChromeBat showcases competitive results across the board, even boasting the highest dSCC score on one outlier chromosome. The fact that a bio-inspired approach performs well has two interpretations. First, the 3D-GRP domain might be best served by using no singular method but instead an ensemble of methods for each task. This is due to the property that different methods appear to have different performances on different instances of the 3D-GRP, even in the same cell line. Secondly, it renders the increase in algorithmic diversity of the studied methods more interesting as certain techniques may dominate portions of the 3D-GRP, but no method will perform best across the entirety of the 3D-GRP. The proposed method seeks to advance the literature on both of these fronts.

### Computation Time

We ran all presented results of ChromeBat by using Intel(R) Xeon(R) CPU E7- 4870 @ 2.40 GHz with 1 Terabyte of RAM and 160 cores. On GM06990 restricted by Ncol at 1 mb, the average computation time per chromosome was 853 s by using the hyperparameters given in Figure 4. In our implementation, these hyperparameters are given in the parametersheavy.txt file (available at https://github.com/OluwadareLab/ChromeBat, accessed on 1 March 2021). However, these parameters call for a search over 30 combinations of α,p, which in implementation becomes 30 concurrent processes. Due to the fact that this could be computationally intense for most local machines, we also provide a parameterslight.txt file that reduces the searched α,p and will only open six concurrent processes.

## 5. Conclusions

We propose the development of the ChromeBat Algorithm as a novel approach to solve 3D-GRP. The domain in general lacks algorithmic diversity; thus, we base our approach in the bio-inspired Bat Algorithm. We find it is capable of state-of-the art performances on real Hi-C cell lines GM12878 and GM06990. This motivates future approaches to consider optimization algorithms that are metaheuristic in nature for the 3D-GRP domain and highlights interest in ensemble models that combine many approaches for chromosomre and genome 3D structural inference.

## Figures and Tables

**Table 1:** The Mann–Whitney Test on Chromosome 1. Each method was used to generate 35 independent structures and the reported number is the P value associated with the Mann–Whitney Test between the column and row methods. For space, the P value has been truncated to five decimals.

Method	LorDG	3Dmax	ShNeigh	HSA	Chromosome3D	ChromeBat
LorDG	n/a	0.00000	0.00000	0.00000	0.00000	0.00000
3Dmax	0.00000	n/a	0.00000	0.00000	0.00000	0.00000
ShNeigh	0.00000	0.00000	n/a	$2.434\times 10^{-2}$	0.00000	0.00000
HSA	0.00000	0.00000	$2.434\times 10^{-2}$	n/a	0.00000	0.00000
Chromosome3D	0.00000	0.00000	0.00000	0.00000	n/a	0.00000
ChromeBat	0.00000	0.00000	0.00000	0.00000	0.00000	n/a

**Table 2:** Fish validation summary. Let function d(X,Y) denote the distance between loci *X* and loci *Y*. A “yes” in the right two columns means that the generated structure is consistent with the FISH data, and a “no” means it is not.

Method	Litigator	*d*(L1,L3) < *d*(L1,L2)?	*d*(L2,L3) < *d*(L2,L4)?
ChromeBat	HindIII	Yes	Yes
	Ncol	Yes	Yes
LorDG	HindIII	No	Yes
	Ncol	No	Yes
HSA	HindIII	No	Yes
	Ncol	No	Yes
3Dmax	HindIII	Yes	Yes
	Ncol	Yes	Yes
Chromosome3D	HindIII	Yes	Yes
	Ncol	No	Yes
ShNeigh	HindIII	No	Yes
	Ncol	No	Yes

**Table 3:** Mann–Whitney U Test on 35 independent structures. We carry out the Mann–Whitney U test between the dSCC of the structures generated with p=0.002 and p=0.004 given in Figure 12 and Figure 13, respectively. Note that all structures are independently generated; thus, each dSCC or observation is independent.

Chromosome	U	*p*
1	0	$3.27\times 10^{-13}$
2	0	$3.27\times 10^{-13}$
3	199	$6.13\times 10^{-7}$
4	0	$3.27\times 10^{-13}$
5	376	$2.7\times 10^{-3}$
6	412	$9.4\times 10^{-3}$
7	492	$7.94\times 10^{-2}$
8	138	$1.29\times 10^{-8}$
9	105	$1.29\times 10^{-9}$
10	110	$1.85\times 10^{-9}$
11	91	$4.68\times 10^{-9}$
12	35	$6.11\times 10^{-12}$
13	70	$9.67\times 10^{-11}$
14	37	$7.19\times 10^{-12}$
15	36	$6.63\times 10^{-12}$
16	0	$3.27\times 10^{-13}$
17	86	$3.23\times 10^{-10}$
18	98	$7.82\times 10^{-10}$
19	24	$2.48\times 10^{-12}$
20	43	$1.16\times 10^{-11}$
21	160	$5.50\times 10^{-8}$
22	251	$1.11\times 10^{-5}$
23	156	$4.24\times 10^{-8}$

## Data Availability

The GM06990 cell lines analysed during the current study are available here: https://github.com/fangzhen-li/ShNeigh/. The GM12878 cell lines analysed during the current study are available in the GSDB repository: http://sysbio.rnet.missouri.edu/3dgenome/GSDB/details.php?id=GM12878. The theoretical yeast chromosome 4 [41] simulated data sets from Adhikari et al. 2016 [19] analysed during the current study are available here: http://sysbio.rnet.missouri.edu/bdm_download/chromosome3d/unzipped/Input/Synthetic/. The helical simulated data sets analysed during the current study are available in the Ouyang Lab repository: https://people.umass.edu/ouyanglab/hsa/downloads.html#Data. The ChromeBat methods generated and analysed during the current study are available in the Oluwadare Lab repository: https://github.com/OluwadareLab/ChromeBat.

## Abbreviations

The following abbreviations are used in this manuscript:

bp	Base pairs;
3D	Three Dimensional;
NGS	Next Generation Sequencing;
TAD	Topologically Associating Domain;
3D-GRP	Three Dimensional Genome Reconstruction Problem;
MDS	Multidimensional Scaling;
LAD	Lamina-Associated Domain;
dSCC	Distance Spearman Correlation Coefficient;
SA	Simulated Annealing;
PSO	Particle Swarm Optimization.
